# Significance of Monoclonal Antibodies against the Conserved Epitopes within Non-Structural Protein 3 Helicase of Hepatitis C Virus

**DOI:** 10.1371/journal.pone.0070214

**Published:** 2013-07-24

**Authors:** Yixin Bian, Shuoxian Zhao, Shaomei Zhu, Jinfeng Zeng, Tingting Li, Yongshui Fu, Yuanzhan Wang, Xin Zheng, Ling Zhang, Wenjing Wang, Baocheng Yang, Yuanping Zhou, Jean-Pierre Allain, Chengyao Li

**Affiliations:** 1 Department of Transfusion Medicine, Southern Medical University, Guangzhou, China; 2 Shenzhen Blood Center, Shenzhen, China; 3 Guangzhou Blood Center, Guangzhou, China; 4 Nanfang Hospital, Southern Medical University, Guangzhou, China; 5 Department of Hematology, University of Cambridge, Cambridge, United Kingdom; University of Navarra School of Medicine and Center for Applied Medical Research (CIMA), Spain

## Abstract

Nonstructural protein 3 (NS3) of hepatitis C virus (HCV), codes for protease and helicase carrying NTPase enzymatic activities, plays a crucial role in viral replication and an ideal target for diagnosis, antiviral therapy and vaccine development. In this study, monoclonal antibodies (mAbs) to NS3 helicase were characterized by epitope mapping and biological function test. A total of 29 monoclonal antibodies were produced to the truncated NS3 helicase of HCV-1b (T1b-rNS3, aa1192–1459). Six mAbs recognized 8/29 16mer peptides, which contributed to identify 5 linear and 1 discontinuous putative epitope sequences. Seven mAbs reacted with HCV-2a JFH-1 infected Huh-7.5.1 cells by immunofluorescent staining, of which 2E12 and 3E5 strongly bound to the exposed linear epitope ^1231^PTGSGKSTK^1239^ (EP05) or core motif ^1373^IPFYGKAI^1380^ (EP21), respectively. Five other mAbs recognized semi-conformational or conformational epitopes of HCV helicase. MAb 2E12 binds to epitope EP05 at the ATP binding site of motif I in domain 1, while mAb 3E5 reacts with epitope EP21 close to helicase nucleotide binding region of domain 2. Epitope EP05 is totally conserved and EP21 highly conserved across HCV genotypes. These two epitope peptides reacted strongly with 59–79% chronic and weakly with 30–58% resolved HCV infected blood donors, suggesting that these epitopes were dominant in HCV infection. MAb 2E12 inhibited 50% of unwinding activity of NS3 helicase *in vitro*. Novel monoclonal antibodies recognize highly conserved epitopes at crucial functional sites within NS3 helicase, which may become important antibodies for diagnosis and antiviral therapy in chronic HCV infection.

## Introduction

More than 170 million people are infected with hepatitis C virus (HCV) worldwide. Approximately 30% of HCV infected individuals spontaneously clear the virus and 70% become chronically infected, the latter being at risk of developing chronic liver disease including liver failure, liver cirrhosis and hepatocellular carcinoma possibly requiring liver transplantation [1].

Nonstructural protein 3 (NS3) provides protease, helicase and NTPase enzymatic activities that play a crucial role in viral replication and constitute a suitable target for antiviral therapy, vaccination and diagnosis in HCV infection. The N-terminal third of NS3 contains a serine-protease domain responsible for processing the nonstructural polyprotein of HCV, while the C-terminal two-thirds encode for an adenosine triphosphatase (ATPase)/helicase capable of unwinding duplex RNA [2]. Previous studies revealed that the NS3 helicase contains immunodominant B-cell epitopes eliciting high levels of antibodies in HCV infected individuals [3]–[6]. The human and murine humoral immune responses to HCV NS3 protein are almost exclusively targeting the ATPase/helicase domain [7], which appears to be serologically reactive during the early phase of HCV infections and is routinely used in clinical diagnostic HCV antibody immunoassays [3].

In recent years, the NS3 helicase has become a popular potential target for exploring inhibitor-helicase interactions in the design of the next generation of HCV NS3 inhibitors [8]. Several highly conserved helicase motifs within HCV NS3 were reported [9]–[12], which interfered with ATP binding or interacted with nucleic acids affecting the helicase function [13], [14]. However, whether those conserved helicase motifs are the epitopes recognized by specific monoclonal antibodies (mAbs) or the binding of mAbs to the conserved motifs of helicase inhibits enzymatic activity remains unknown. This study explored extensively monoclonal antibodies to antigenic epitopes of NS3 helicase and their potential applications for diagnosis and antiviral drugs in HCV infection.

## Materials and Methods

### Ethics Statement

All animal care and procedures were in accordance with national and institutional policies for animal health and well-being. Mouse experimentation and sample collection were approved by Southern Medical University (SMU) Animal Care and Use Committee (permit numbers: NFYY-2008-043). Mouse surgery was performed under anesthesia, and all efforts were made to minimize suffering of animals.

### Recombinant HCV NS3

The fragment of NS3 cDNA was isolated from HCV genotype 1b strain (GenBank accession No. JN870283). The truncated recombinant NS3 helicase (T1b-rNS3) covering the functional part of amino acid (aa) sequence (aa 166–433 for NS3 or aa 1192–1459 for whole protein) was expressed in *E. coli* with pET-32a vector (Novagen, Merck KGaA, Darmstadt, Germany). T1b-rNS3 was produced in *E. coli* by inducing for 4 hrs with 1 mM Isopropyl-1-thio-D-galactopyloranoside (IPTG) at 37°C. The cells were harvested and sonicated. Soluble T1b-rNS3 was purified by Ni-NTA agarose according to the manufacturer’s instructions (GE Healthcare, Milwaukee, Wisconsin, USA) and analyzed by Sodium Dodecyl Sulfate–Polyacrylamide Gel Electrophoresis (SDS-PAGE). The purity of T1b-rNS3 was over 90%. The full-length recombinant NS3 of HCV genotype 1b (FL1b-rNS3, aa 1–631 or aa 1027–1657) was expressed with lentiviral construct pTY-CMV in 293T cells [15]. The full-length recombinant NS3 of HCV genotype 4b (FL4b-rNS3) produced in *E. coli* was purchased from a company (CUSABIO, Wuhan, China). The purity of FL4b-rNS3 was over 95%.

### Peptides

A panel of 47 peptides was commercially synthesized (Chinese Peptide Company, Hangzhou, Zhejiang, China) (Table 1). Twenty-nine of 16mer peptides with 7mer overlap were designated as P01 to P29 spanning 268 amino acids of HCV NS3 between aa 1192 and 1459. Twelve 6–11mer peptides from P05 and P21 were shortened and designated as P0501 to P0506 or P2101 to P2106. Three peptides were designated as VatP2101-03 corresponding to P21 derived from HCV variants with aa substitutions. Two peptides were designated as GP05′ and GP21′ corresponding to HCV P05 or P21 sequence derived from GB virus C (GBV-C). One peptide derived from BP26 protein of *Brucella melitensis* strain was used as a negative control (NC). All peptides were >90% purity.

**Table 1:** Overlapping peptides of HCV NS3 helicase (aa 1192–1459).

Peptide	Sequence	Aa position (NS3/whole)
Group A		
P01	AVDFIPVESMETTMRS	166–181/1192–1207
P02	METTMRSPVFTDNSSP	175–190/1201–1216
P03	FTDNSSPPAVPQTFQV	184–199/1210–1225
P04	VPQTFQVAHLHAPTGS	193–208/1219–1234
P05	LHAPTGSGKSTKVPAA	202–217/1228–1243
P06	STKVPAAYAAQGYKVL	211–226/1237–1252
P07	AQGYKVLVLNPSVAAT	220–235/1246–1261
P08	NPSVAATLGFGAYMSK	229–244/1255–1270
P09	FGAYMSKAHGTDPNIR	238–253/1264–1279
P10	GTDPNIRTGIRTITTG	247–262/1273–1288
P11	IRTITTGAPITYSTYG	256–271/1282–1297
P12	ITYSTYGKFLADGGCS	265–280/1291–1306
P13	LADGGCSGGAYDIIMC	274–289/1300–1315
P14	AYDIIMCDECHSTDST	283–298/1309–1324
P15	CHSTDSTTILGIGTVL	292–307/1318–1333
P16	LGIGTVLDQAETAGAR	301–316/1327–1342
P17	AETAGARLVVLATATP	310–325/1336–1351
P18	VLATATPPGSVTVPHP	319–334/1345–1360
P19	SVTVPHPNIEEVALSN	328–343/1354–1369
P20	EEVALSNTGEIPFYGK	337–352/1363–1378
P21	EIPFYGKAIPIETIKG	346–361/1372–1387
P22	PIETIKGGRHLIFCHS	355–370/1381–1396
P23	HLIFCHSKKKCDELAA	364–379/1390–1405
P24	KCDELAAKLSGLGLNA	373–388/1399–1414
P25	SGLGLNAVAYYRGLDV	382–397/1408–1423
P26	YYRGLDVSVIPTSGDV	391–406/1417–1432
P27	IPTSGDVVVVATDALM	400–415/1426–1441
P28	VATDALMTGFTGDFDS	409–424/1435–1450
P29	FTGDFDSVIDCNTCVT	418–433/1444–1459
Group B		
P0501	PTGSGKSTKV	205–214/1231–1240
P0502	PTGSGKSTK	205–213/1231–1239
P0503	TGSGKSTKV	206–214/1232–1240
P0504	PTGSGKST	205–212/1231–1238
P0505	TGSGKSTK	206–213/1232–1239
P0506	GSGKST	207–212/1233–1238
P2101	EIPFYGKAIPI	346–356/1372–1382
P2102	EIPFYGKAIP	346–355/1372–1381
P2103	EIPFYGKAI	346–354/1372–1380
P2104	IPFYGKAIP	347–355/1373–1381
P2105	EIPFYGKA	346–353/1372–1379
P2106	PFYGKAIP	348–355/1372–1381
Group C		
VatP2101	GE**V**PFYGKAIPLEYIR	Corresponding to P21
VatP2102	GEIPFYG**R**AIPLALIK	Corresponding to P21
VatP2103	GEIPFYGKA**L**PLAAIK	Corresponding to P21
Group D		
GP05’	LFMPTG**A**GKST**R**VPLE	Corresponding to P05
GP21’	GEIPFYG**HG**IPLERMR	Corresponding to P21
Group E		
NC	NIQPIYVYPDDKNNLK	

Five groups of synthetic peptides derived from NS3 (1192–1459) of HCV genotype 1b (Genebank accession number AFC36922) are listed in the Table. Group A, 29 of 16mer peptides with 7mer overlapping spanning 268 amino acids of HCV NS3 helicase between aa 1192 and 1459. Group B, 6–10mer peptides are shortened from P05 or P21. Group C, peptides with an amino acid substitution from the region corresponding to P21 are derived from other genotypes of HCV variants. Group D, peptides are derived from the region of GBV-C corresponding to P05 or P21. Group E, negative control peptide (NC) is derived from *Brucella melitensis* BP26 protein. Aa, amino acid position in NS3 or whole ORF protein. Letters in the bold with underline indicate the amino acid substitution aligned with P21 or P05.

### Monoclonal Antibody Production

Three 6-weeks old BALB/c female mice were immunized with T1b-rNS3 antigens three times at 2-week intervals. The immunized spleen cells were fused with SP2/0 myeloma cells with PEG 4000 (Sigma-Aldrich, St Louis, Missouri, USA) [16]. Single hybridoma cells were cloned by limiting dilution. MAb isotyping was performed by IsoQuick Strips (Sigma-Aldrich, St Louis, Missouri, USA). MAbs were purified by Protein G column chromatography (Millipore, Bellerica, MA, USA). One mAb (IgG1 kappa) to recombinant BP26 of *B. melitensis* M5–90 was used as an unrelated negative control.

### Virus Cell Culture and Native NS3

HCV was generated by transfection of an infectious RNA of HCV genotype 2a (JFH-1) to Huh7.5.1 cells (provided by Dr Yuanping Zhou). HCV was inoculated to the fresh Huh7.5.1 cells for viral culturing and passaging. The NS3 produced in HCV JFH-1 infected cells (called native NS3) was detected with mAbs. Dengue virus serotypes 2 (DV-2) infected vero cells (provided by Dr Wei Zhao) were tested for mAb’s cross-reactivity.

### Peptide-ELISA

Nunc Immuno microtiter plates coated with 5 µg/ml of peptides were used to react with hybridoma supernatants as described previously [16]. Goat anti-mouse IgG and IgM horseradish peroxidase (HRP)-conjugate (Rockland Immunochemicals Corp, Boyertown, Pennsylvania, USA) was used as secondary antibody, and 3,3′−5,5′ tetramethylbenzidine (TMB) was used as colorimetric substrate. The developed color was measured with a microplate reader with a 450 nm filter. An unrelated peptide derived from *B. melitensis* was used as negative control.

### Western-blot

NS3 protein from *E. coli* or extract from cell lysate was electrophoresed on SDS-PAGE and transferred onto PVDF membranes (Millipore, Billerica, Massachusetts, USA). Protein-bound membranes were saturated with the supernatants of mAb cultures, detected by goat anti-mouse IgG and IgM HRP-conjugate and finally visualized by adding immunochemiluminescence reagent (ECL, Millipore, Billerica, Massachusetts, USA). The un-transfected or un-infected cells were used as negative cell controls.

### Immunofluorescence Staining (IFS)

FL1b-rNS3 expressing 293T cell, HCV JFH-1 infected Huh7.5.1 cell or Dengue virus infected vero cell cultures in 96-well plates were fixed in 100% methanol. Cells were individually incubated with mAbs as primary antibodies, followed by incubation with Alexa Fluor 594-conjugated goat anti-mouse secondary IgG (H+L) (Invitrogen China Limited, Guangzhou, China**)**. MAb C7–50 reactive to HCV core protein (Abcam, Cambridge, UK) was used as positive control of primary antibody. An un-related mAb to *B. melitensis* was used as negative control. The untransfected or uninfected cells were used as negative cell controls. Reactive mAbs to cells were examined with a Nikon Labophot photomicroscope with the epifluorescence attachment EF-D (Nikon, Garden City, NY, USA).

### Epitope Amino Acid Sequence Alignment

Amino acid sequences corresponding to the regions of epitopes within HCV NS3 helicase and relevant flaviruses were randomly selected from GenBank database. The accession numbers are as follows: AFC36922 (HCV 1b), ABL96700 (HCV 1a), AAU89634 (HCV 2a), AAF59945 (HCV 2b), ADY38590 (HCV 3a), ABB89469 (HCV 3b), YP_001469632 (4a), ABU68271 (HCV 4f), AAC61696 (HCV 5a), ABP88847 (HCV 6l), ABB84854 (HCV 6k), ABP88845 (HCV 6p), CAD21957 (GB virus B, GBV-B), BAA22479 (GB virus C, GBV-C), ACS31924 (Dengue virus 1, DV-1), AAK67712 (DV-2), AEA50923.1 (DV-3), AEX09561 (DV-4), AAA81554 (Japanese encephalitis virus, JEV), ADT91913 (West Nile virus, WNV) and AEQ35299 (Yellow fever virus, YFV).

### HCV Plasma and Detection

Donor blood samples were collected from Shenzhen and Guangzhou blood centers in Guangdong province [17]. The written informed consents were obtained from all blood donors. Plasma samples used in this study were approved by Southern Medical University (SMU) Nan Fang Hospital Medical Ethics Committee (permit numbers: NFYY-2009–23) in accordance with national and institutional policies for medical ethics. Anti-HCV reactive plasma samples negative for both HBsAg and anti-HIV were confirmed when reactive with at least two domestic and international commercial EIAs. Samples confirmed anti-HCV positive and RNA positive (Ab+/RNA+) were classified as chronic infection. Those confirmed antibody positive RNA negative (Ab+/RNA-) were classified as recovered infection [17]. The cutoffs of peptides reactive to negative control samples were calculated as mean+2SD with 95% confidence interval (CI) in ELISA. Anti-HCV reactivity was reported as mean of signal to cut-off ratio (S/CO).

### Fluorescence Helicase Activity Assay

The fluorescence helicase unwinding assay using dsDNA substrate was performed as described [18]. DNA oligonucleotides were labeled with Fluorescein amidite (FAM) and Black Hole Quencher 1 (BHQ1) (Invitrogen China Limited, Guangzhou, China). The sequences of fluorescent or quencher oligonucleotide strands are 5′-(FAM)TAGTACCGCCACCCTCAGAACCTTTTTTTTTTTTTT-3′ and 5′-GGTTCTGAGGGTGGCGGTACTA(BHQ1)-3′, respectively. The stock of dsDNA substrates was prepared to a final concentration of 10 µM in 20 mM Tris-HCl (pH 7.5) by combining single strands of donor and complementary oligonucleotides in a 1∶3 molar ratio, heating at 95°C, then slow cooling to 65°C 15 min, 55°C 15 min and 30°C for 2 hours. The helicase unwinding assay was performed in 30 mM Tris–HCl (pH 7.5), 5 mM MgCl2, 0.075% Triton X-100, 100 nM dsDNA substrate, 1.5 mM ATP, and 1 µM capture strand (5′-CTACTACCCCCACCCTCACAACC-3′) in 50 µl of reaction volume. The unwinding reaction was started by adding HCV NS3 helicase (0–100 nM) and was carried out at 30°C for 90 min using a quantitative PCR M×3005P (Agilent Technologies, Waldbronn, Germany). Fluorescence intensity was recorded every 2 min. The helicase activity was calculated as the initial reaction velocity from the linear part of the progress curve using the linear regression method. A linear equation Y = A+BX was fitted to the experimental data, where Y is the helicase activity expressed in fluorescence units, X the reaction time, and B the slope of the initial velocity curve.

### Statistical Analysis

Data analysis was performed using the SPSS software version 13.0. Pearson Chi-Square test was used for comparison of difference between the peptides reacting with chronic and resolved HCV infected sample groups. One-way ANONA test and Dunnett’s T3 were used to compare the difference of NS3 helicase activity between experimental and control groups. *P* value <0.05 was considered significant.

## Results

### Epitope Classification of Monoclonal Antibodies to HCV NS3

**Figure 1:**
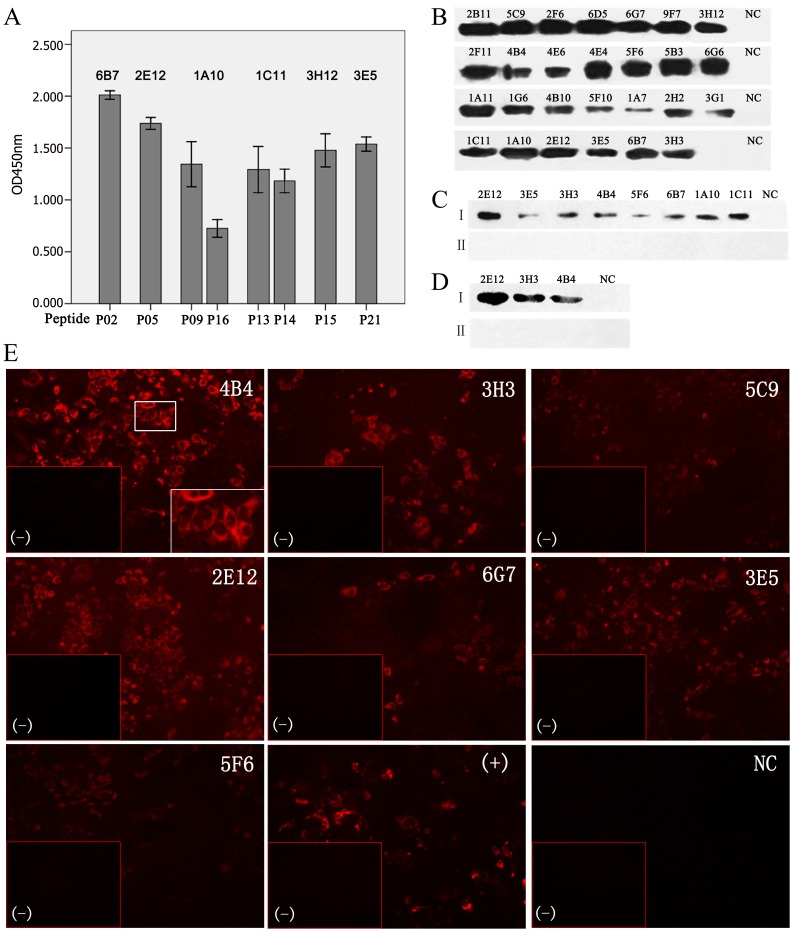
Reactivity of mAbs to peptides and proteins of NS3 helicase. MAbs reacted with 16mer peptides in Peptide-ELISA (A), denatured T1b-rNS3 in Western Blot (B), denatured FL1b-rNS3 expressing 293T cells (C. I) and denatured native NS3 of HCV JFH-1 (2a) infected Huh7.5.1 cells (D. I) in Western Blot, and non-denatured native NS3 of HCV JFH-1 infected Huh7.5.1 cells in IFS (E). C. II and D. II, un-transfected 293T or un-infected Huh7.5.1 cell controls, respectively; NC (negative control), an un-related mAb to BP26 protein of *B. melitensis*; (+), mAb C7–50 to HCV core as positive control; (−), un-infected Huh7.5.1 cells as negative control.

A total of 29 mAbs were screened for reactivity with T1b-rNS3 by ELISA. To classify epitope recognition, mAbs were tested for reactivity with peptides, denatured and non-denatured viral proteins. In contrast to recombinant NS3 proteins T1b-rNS3 and FL1b-rNS3 expressed from *E. coli* or 293T cells, the NS3 protein produced from HCV JFH-1 infected Huh7.5.1 cells was defined as native NS3 of viral proteins. Among 29 16mer overlapping peptides (Table 1), six mAbs reacted with 8 of 16mer peptides (Figure 1A). MAb 1C11 recognized peptides P13 and P14 that shared a putative epitope sequence, while mAb 1A10 bound to two non-overlapping peptides P09 and P16 that might constitute a discontinuous epitope. Altogether, using synthetic peptides as target, mAbs recognized five putative linear and 1 discontinuous epitope sequences from peptides. By Western-blot (WB) analysis, twenty-seven mAbs reacted with the denatured T1b-rNS3 expressed from *E. coli* (Figure 1B) and eight mAbs reacted with the denatured FL1b-rNS3 expressed from 293T cells (Figure 1C), while only three of them cross-reacted with the denatured native NS3 produced from HCV JFH-1 (2a) infected Huh7.5.1 cells (Figure 1D). Ten mAbs reacted with the non-denatured FL1b-rNS3 in 293T cells, and seven (6 of 10 and 1 extra) cross-reacted with the non-denatured native NS3 in HCV JFH-1 infected cells in IFS (Figure 1E). The detection for NS3 was observed in the cytoplasma of HCV infected cells, in which over 50% HCV infected cells were strongly stained by mAbs 4B4, 2E12 and 3E5 (Figure 1E). None of mAbs was detected for reactivity with un-transfected 293T or un-infected Huh7.5.1 cell controls by WB and IFS. According to mAb recognition for peptide, denatured or non-denatured antigen, NS3 helicase epitopes were classified as six linear, three semi-conformational and three conformational, respectively (Table 2).

**Table 2:** Classification of mAbs reacting with HCV NS3 helicase.

MAb	Isotype	ELISA/WB(T1b-rNS3)	ELISA (peptide)	WB (FL1b-rNS3/native NS3 2a)	IFS (FL1b-rNS3/native NS3 2a)	Epitope type (aa position)
2E12	IgG1 (k)	+/+	+	+/+	+/+	L (1231–1238)
3E5	IgG2a (k)	+*/+	+	+/−	+/+	L (1373–1380)
4B4	IgG1 (k)	+/+	−	+/+	+/+	SC
3H3	IgG1(k)	+/+	−	+/+	+/+	SC
5C9	IgG1 (k)	+/+	−	−/−	−/+	C
5F6	IgG2a (k)	+/+	−	+/−	+/+	SC
6G7	IgG1(k)	+/+	−	−/−	+/+	C
6B7	IgM (k)	+/+	+	+/−	+/−	L (1201–1216)
1A10	IgG2b (k)	+/+	+	+/−	+/−	L (1264–1279, 1327–1342)
1C11	IgG1 (k)	+/+	+	+/−	+/−	L (1300–1315)
3H12	IgG1 (k)	+/+	+	−/−	−/−	L (1318–1333)
1A7	IgG1	+/+	−	−	+/−	C
15 clones	NT	+/+	−	−	−	UN
2 clones	NT	+/−	−	−	−	UN

Aa, amino acid position; L, linear; SC, semi-conformational; C, conformational; UN, un-classified; NT, not tested; the native NS3 indicates NS3 produced from HCV JFH-1 infected cells;

* indicates mAb 3E5 reactive to both T1b-rNS3 with 347I and FL4b-rNS3 with 347V in ELISA.

### Fine Mapping for the Linear Epitopes of HCV NS3

**Figure 2:**
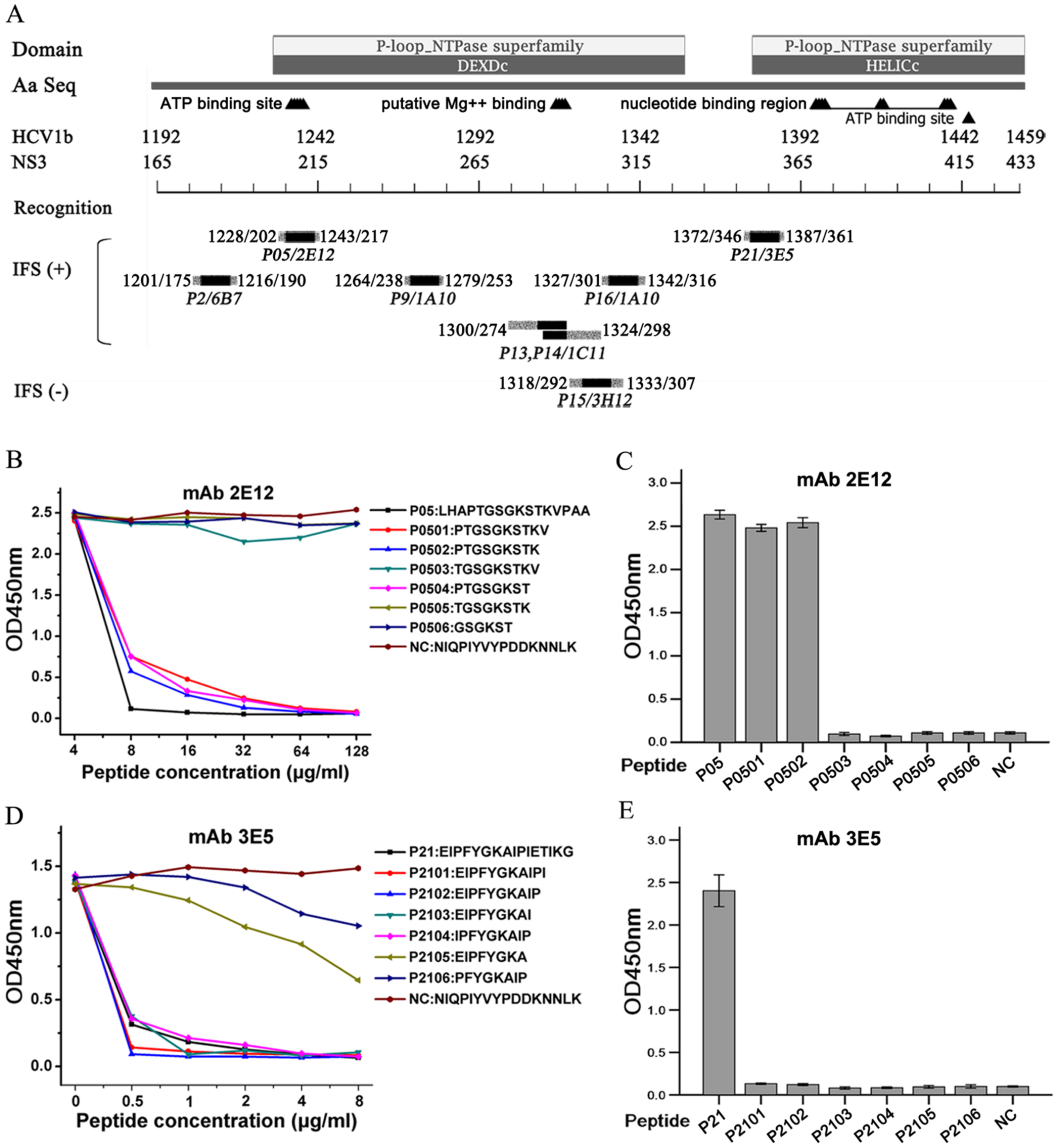
Epitope mapping for NS3 helicase recognized by mAbs. (A) Peptides reactive to mAbs are localized to the corresponding aa sequences of NS3 helicase from HCV 1b. Aa sequence position is indicated by numbering either whole or NS3 protein sequence. Recognition of mAb to peptide is indicated by names of peptide and mAb. Recognitions of mAbs reactive to epitopes of FL1b-rNS3 expressing 293T cells are divided into positive (+) and negative (−) groups by IFS. (B and D) The binding of mAb 2E12 or 3E5 (0.5 µg/ml) to P05 or P21 coated plate was inhibited by the shortened peptides (P0501–0506 or P2101 to P2106). (C and E) MAb 2E12 or 3E5 bound to the plate coated with shortened peptides derived from P05 or P21. An un-related peptide from *B. melitensis* BP26 was used as a non-inhibitory control (NC) in Peptide-ELISA.

Six linear epitope sequences are localized within the amino acid residues 1192–1459 of HCV NS3 helicase (Figure 2A). According to the intact NS3 protein recognized by IFS, five epitopes are exposed and one is unexposed in FL1b-rNS3 expressing cells. Two mAbs cross-reacted with the exposed linear epitopes of native NS3 in HCV JFH-1 (2a) infected cells, mAb 2E12 reacting with the sequence corresponding to the ATP binding site, and mAb 3E5 with a sequence close to the nucleotide binding region of NS3 helicase.

In order to further define those two exposed linear epitopes at critical function sites of helicase, peptides P05 or P21 were shortened and tested for direct and competitive binding to mAbs 2E12 and 3E5 in Peptide-ELISA, respectively. The binding of mAb 2E12 to P05 (16 mers) coated plate was inhibited by the shorter peptides P0501 (10 mers), P0502 (9 mers), P0504 (8 mers) and P05 itself, but not by control peptide (NC) (Figure 2B). Antibody 2E12 reacted only with P0501 or P0502 coated plates (Figure 2C), suggesting that the minimal amino acid residues of the linear epitope (EP05, PTGSGKSTK) recognized by mAb 2E12 was located at position 1231–1239 of the whole polyprotein or 205–213 of the NS3 protein of HCV 1b. The binding of mAb 3E5 to P21 (16 mers) coated plate was competitively inhibited by the shorter peptides P2101 (11 mers), P2102 (10 mers), P2103 (9 mers), P2104 (8 mers) and P21 itself, but not by control peptide (Figure 2D). Discrepantly, mAb 3E5 did not bind any shorter peptide coated plate (Figure 2E), suggesting that the core motif ^1373^IPFYGKAI^1380^ within the longer amino acid residues of epitope P21 (EP21, ^1372^EIPFYGKAIPIETIKG^1387^) could be recognized by 3E5 in the free status but not in the fixed form on the plate.

### Specificity of HCV NS3 Epitopes

**Figure 3:**
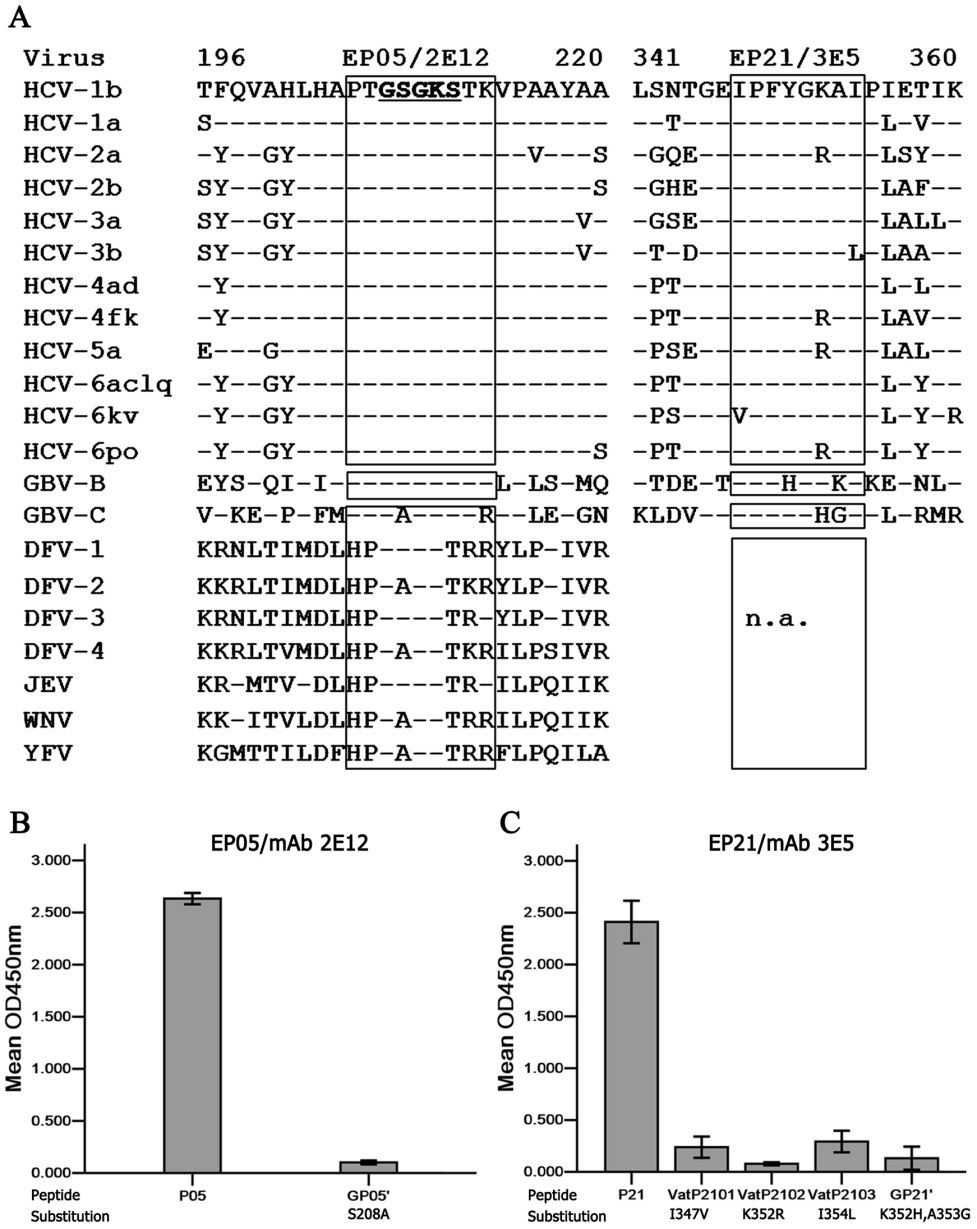
Specificity of two linear epitopes within NS3 helicases of HCV and other flaviviruses. (A) Analysis of amino acid sequence corresponding to EP05 and EP21 regions within NS3 helicase cross HCV genotypes and other flaviviruses. Aa sequences (one-letter code) to epitopes of HCV genotype 1b are presented on the top. Positions at the beginning and end of sequences are indicated by numbers. Identities with the lead sequence are indicated by dashes. Representative sequences are retrieved from Genebank Database. Triangles labeling with EP05/2E12 or EP21/3E5 on the top indicate epitope sequences or corresponding sequences for mAb’s recognition. The aa residues GSGKS underlined in bold indicate the ATP binding site of motif I (Walker A) within NS3 helicase. n.a. indicates no corresponding sequence available from those viruses. (B and C) Reactivity of mAb 2E12 or 3E5 with mutant peptide corresponding to the defined epitope sequence in Peptide-ELISA.

To analyze diversity or similarity of the two identified linear epitopes, the corresponding amino acid consensus sequences from each genotype of HCV and other human flaviviruses were compared with the sequences of epitope EP05 or core motifs of epitope EP21 within NS3 helicases (Figure. 3A). EP05 sequence ^205^PTGSGKSTK^213^ recognized by mAb 2E12 was totally conserved across HCV genotypes, which was also present in the non-human flavivirus GBV-B. The core motif ^347^IPFYGKAI^354^ of EP21 recognized by mAb 3E5 was highly conserved in genotype 1, 2b, 3a and 6a, c, l and q. Amino acid differences were found in genotype 3b (I354L), genotype 4f, 4r, 5a, 6p and 6o (K352R) and genotype 6k and 6v (I347V) (Figure 3A). MAb 2E12 did not react with peptide GP05’ when S208A and K213R substitutions corresponding to the GBV-C sequence were present (Figure 3B), suggesting that the amino acid Serine 208 was critical for epitope EP05 specificity to HCV. MAb 3E5 did not react with peptide Vat2101 (I347V) or peptide Vat2102 (K352R), weakly reacted with peptide VatP2103 (I354L) corresponding to epitope EP21 derived from HCV variants (Figure 3C), but strongly reacted when substitution I347V or K352R was present in full-length recombinant NS3 (FL4b-rNS3, genotype 4b) or HCV JFH-1 infected cells (genotype 2a), respectively (Table 2 and Figure 1E). MAb 3E5 did not bind to the corresponding peptide GP21’ (K352H and A353G) derived from GBV-C (Figure 3C). Both mAbs 2E12 and 3E5 were non-reactive with Dengue virus infected cells by immunofluorescence staining.

### Reactivity of Linear Epitope Peptides with HCV Infected Plasmas

To evaluate the potential capacity of the two highly conserved epitopes (P05 and P21) to elicit antibody response in HCV infected individuals, 136 HCV infected, 42 HBsAg+ and 128 healthy plasmas from Chinese blood donors were tested for reactivity with epitopes EP05 or EP21 mimicked by peptides P05 and P21 using Peptide-ELISA (Table 3). Of 306 plasmas, 86 were from individuals with chronic HCV infection, 50 from individuals who spontaneously resolved HCV infection, 42 from individuals with HBsAg+ and 128 non-infected individuals (Ab−/RNA-). In plasmas from chronically infected individuals, reactivity to epitope peptides P05 and P21 was 79.1% and 59.3%, respectively. Combined two peptides reacted with 82.6% of samples and no obvious difference between genotypes was observed. Lower prevalence of reactivity was found in resolved cases (30–58% for both peptides), which was significantly different from reactivity in chronic cases (*P*<0.001). Specificity was above 97.6% for each peptide but false reactions cumulated.

**Table 3:** Reactivity of epitope peptides with HCV infected plasmas.

HCV samples	P05 (EP05)	P21 (EP21)	P05+P21
Chronic Nb	86	86	86
Nb+(%)	68 (79.1)	51 (59.3)	71 (82.6)
Nb+/genotyped	52/64 (81.3)	39/64 (60.9)	54/64 (84.4)
Genotype 1	26/30 (86.7)	14/30 (46.7)	26/30 (86.7)
Genotype 2	7/9 (77.8)	7/9 (77.8)	8/9 (88.9)
Genotype 3	6/10 (60.0)	6/10 (60.0)	7/10 (70.0)
Genotype 6	13/15 (86.7)	12/15 (80.0)	13/15 (86.7)
Resolved Nb	50	50	50
Nb+(%)	29 (58.0)	15 (30.0)	29 (58.0)
*P* value	<0.001	<0.001	<0.001
Total Nb	136	136	136
Nb+(%)	97 (71.3)	66 (48.5)	100 (73.5)
HBsAg+Nb	42	42	42
Nb false+(%)	1 (2.4)	1 (2.4)	1 (2.4)
Healthy Nb	128	128	128
Nb false+(%)	3 (2.3)	3 (2.3)	6 (4.7)

### Inhibition of HCV NS3 Helicase Activity by Monoclonal Antibody

**Figure 4:**
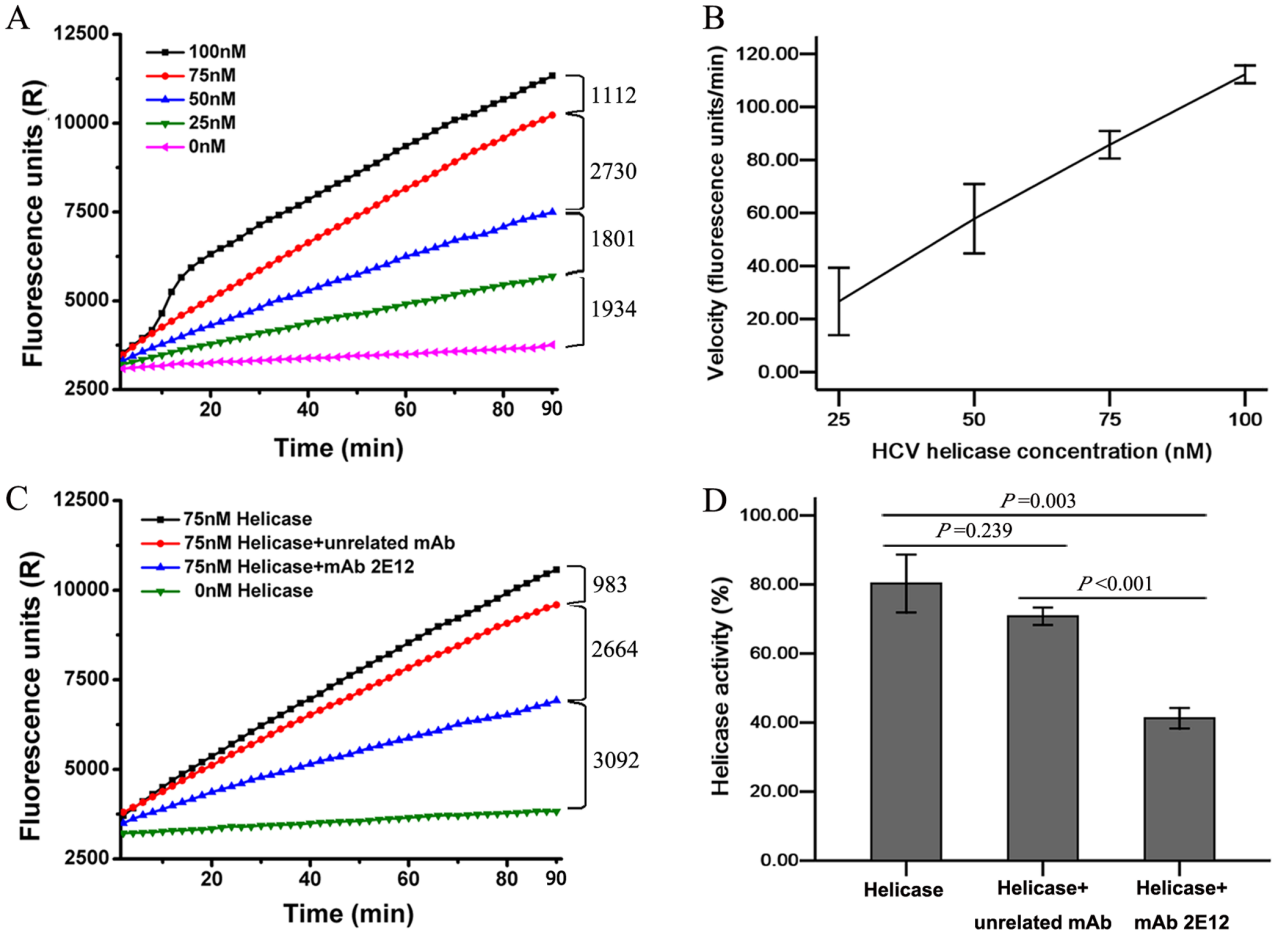
Unwinding activity of HCV NS3 helicase inhibited by mAb 2E12. (A) A representative of DNA oligonucleotides labeled with Fluorescein amidite (FAM) from unwound double-strand DNA substrates at various concentrations of NS3 helicase of HCV (FL4b-rNS3) in unwinding reactions. (B) The slope of the initial velocity curve calculated from 4 representative tests with mean±SD. (C) A representative of unwinding reactions with 75 nM NS3 helicase and 1 µg/ml mAb 2E12 or an unrelated control mAb to *B. melitensis*. (D) Percentages for unwinding activity measured as velocity from 4 representative tests of unwinding reactions with NS3 helicase and mAbs.

MAb 2E12 recognizes an epitope covering the ATP binding site in domain 1 of the helicase raising the possibility of functional interference of this antibody in the helicase function. To investigate this possibility, mAb 2E12 and an unrelated control were added to a full-length recombinant protein HCV NS3 genotype 4b (FL4b-rNS3) in an unwinding assay. FL4b-rNS3 exhibited helicase unwinding activity (Figure 4A and B), that was inhibited by mAb 2E12 but not by an irrelevant mAb control (Figure 4C and D). Approximately 50% reduction in unwinding activity by this mAb was observed (Figure 4D) (*P*<0.001), suggesting that 2E12 may play a role as HCV helicase inhibitor.

## Discussion

ATPase/helicase is the major antigen recognized by B-cells [7]. In previous studies, few monoclonal antibodies to NS3 helicase were characterized mostly for conformational epitopes. A human monoclonal antibody CM3.B6 recognized a major epitope located at the minimal residues 1378–1443 within c33c [4]. A murine mAb ZX10 recognized a discontinuous epitope of NS3 helicase domain, encompassing residues 1371 to 1382 [5]. However, monoclonal antibodies to NS3 helicase have been neither generated nor explored extensively for epitope identification and impact of monoclonal antibodies on its enzymatic function in HCV infection.

In this study, 10 mAbs recognized FL1b-rNS3 expressing cells and seven mAbs cross-reacted with native NS3 in HCV JFH-1 (2a) infected cells by IFS, of which mAbs 2E12 and 3E5 bound to the exposed linear epitopes and three and two other mAbs reacted with semi-conformational or conformational epitopes, respectively (Table 2). MAb 2E12 binding to linear epitope EP05 was fine mapped to minimal aa residues ^1231^PTGSGKSTK^1239^, while mAb 3E5 recognizing core motif of EP21 was reduced to aa residues ^1373^IPFYGKAI^1380^ at presence of flexible form. The novel epitope EP05 is located in motif I of domain 1 within NS3 helicase of HCV, which covers the ATP binding site (aa residues GSGKS) critical for the enzymatic activity of NS3 helicase [9], [11], [12]. The core motif of epitope EP21 recognized by mAb 3E5 is accurately defined as 4 mers shorter than a discontinuous epitope constituted partly with aa residues ^1371^GEIPFYGKAIPL^1382^ recognized by mAb ZX10 previously [5]. Epitope EP21 is located between motif III and motif IV close to the nucleotide-binding region of domain 2 within NS3 helicase [10], [11]. The binding of mAb 2E12 or 3E5 to these critical sites could putatively affect the enzymatic function of NS3 helicase in HCV replication.

Detection of NS3 from cells or tissues is a critical evidence for interpretation of HCV pathogenicity. In clinical investigations, NS3 positive cells were significantly detected from liver, brain or kidney tissue of HCV-infected patients [19]–[21]. The rate for visualizing HCV infected cells mainly relied on the efficacy of monoclonal antibodies binding to NS3. However, rare mAbs were available for this purpose due to the high diversity of HCV genotypes. In this study, mAb 2E12 and 3E5 strongly reacted with epitope EP05 or EP21, of which EP05 sequence was completely conserved in all HCV genotypes (Figure 3A), while EP21 was highly conserved with few substitutions in several genotypes of HCV [22]. Both mAbs 2E12 and 3E5 had high specificity for the conserved epitopes within NS3 helicase of HCV, which did not react with other human flaviviruses including GB virus C (GBV-C) that carried a sequence differing by a single substitution (S208A) from epitope EP05. Interestingly, even though mAb 3E5 did not bind to the linear peptides Vat2101 and Vat2102 with substitution I347V or K352R corresponding to the core motif sequence of epitope EP21 (Figure 3C), it still strongly reacted with the full-length recombinant NS3 protein (FL4b-NS3) or HCV JFH-1 (2a) infected cells carrying the identical mutants (Table 2 and Figure 1E), suggesting that the substitution of I347V or K352R within the core motif of EP21 did not alter the epitope conformation presented in NS3 helicase proteins but differed in linear peptide form. MAb 2E12 recognized an identical epitope (EP05) from a non-human hepatovirus GBV-B infected hepatocytes in marmosets (data not shown), which was used as an attractive surrogate animal model for HCV infection [23]. The data presented here suggests that mAbs 2E12 and 3E5 may take an important place among the major antibodies used for detection of HCV infected cells. Mimicking the novel epitope EP05 (containing the ATP binding site), broadly reactive monoclonal antibodies to the corresponding epitopes might be produced for detections of human flaviviruses such as Dengue, West Nile, Japanese encephalitis and Yellow fever viruses.

A number of epitope peptides derived from NS3 helicase were selected for reacting with sera of HCV infected patients [4], [24], which did not include the EP05 or EP21 epitope peptides. In the present study, peptides P05 and P21 reacted strongly with 79% or 59% chronic HCV infected plasmas, which were more active than 58% or 30% weakly reactive resolved HCV infected plasmas from blood donors (*P*<0.001). The results indicated that both peptides contained immunodominant epitopes eliciting antibody response mostly in chronic HCV infection.

Targeting of NS3 helicase is becoming a promising approach to inhibit viral replication in HCV infection [11]. Recombinant antibodies to NS3 helicase has been introduced into hepatocytes by viral vectors, which effectively inhibited HCV replication [5], [25]–[27]. The most critical residues for ATP binding arise from the Walker A and B motifs within NS3 helicase of HCV, in which the Walker A motif forms a phosphate binding loop (P-loop) with the conserved Lysine (210 K) likely contacting the γ phosphate of ATP [9], [11]. The ATP binding site of NS3 helicase with totally conserved amino acids played a critical role in sustaining unwinding activity [28], is the precise linear epitope reactive with mAb 2E12. In the present study, mAbs 2E12 interacted to win the 50% dysfunction of unwinding activity for FL4b-rNS3 *in vitro*, suggesting that 2E12 might have potential for inhibiting the enzymatic activity of NS3 helicase. In a prospective study, recombinant mAb 2E12 within a viral vector will be transduced into Huh7.5.1 cells for further evaluating its intracellular capacity for inhibiting helicase activity and HCV replication *in vivo*.

## Conclusions

We generated two mAbs 2E12 and 5E5, which specifically bound to finely defined and highly conserved epitopes of HCV NS3 helicase, respectively. The binding of mAbs to the ATP binding site at motif I (Walker A) of domain 1 or the proximity to nucleotide binding region of domain 2 within NS3 helicase might affect the enzymatic activity of helicase in HCV replication. The present data suggested that mAbs 2E12 and 3E5 might carry considerable potential for diagnostics and antiviral therapy of chronic HCV infection.
